# Preheating Modeling of Forming Region and Design of Electrode Structure During Integral Electric Hot Incremental Forming

**DOI:** 10.3390/nano15090698

**Published:** 2025-05-06

**Authors:** Zhengfang Li, Lijia Liu, Jiangpeng Song, Shuang Wu, Li Liu, Xinhao Zhai

**Affiliations:** School of Mechanical and Electrical Engineering, Kunming University, Kunming 650214, China

**Keywords:** incremental sheet forming, hot forming, heat flux, electrode design, thermal simulation

## Abstract

Recently, integral electric hot incremental forming technology has been proposed to form hard-to-form sheet metals and to eliminate some defects obtained through the local heating method via current, such as inhomogeneous temperature distribution, arc burns for the sheet and the tool, unsuitability for multistage forming, etc. However, the simulation of integral electric hot incremental forming involves coupled electro-thermal-mechanical analysis, which is difficult through existing simulation software. Meanwhile, the effect of the electrode structure on temperature distribution is not clear; therefore, a preheating flux model for Joule heat was proposed to simulate the temperature distribution of Ti-6Al-4V titanium alloy sheet in this work, which could simplify the coupled electro-thermal-mechanical analysis to the coupled thermal–mechanical simulation. Meanwhile, the effect of the electrode section and length on the temperature distribution was analyzed in detail, and then a design criterion for the electrode length was obtained during integral electric hot incremental forming.

## 1. Introduction

Incremental sheet forming, as a flexible and green process, can quickly fabricate products with lower costs and shorter manufacturing cycles, which is especially suitable for small batch parts [1,2]. Therefore, some fields, such as aeronautics, product research and development, and medicine, are gradually applying this technology to the production of corresponding products [3,4,5]. In recent decades, the forming process has mainly included two branches, namely cold forming and hot forming. For the former, some forming characteristics of certain metallic materials, such as deformation behavior, surface roughness formability, forming force, and microstructure, have been analyzed in detail due to cold forming’s earlier start [6,7,8].

**Figure 1:**
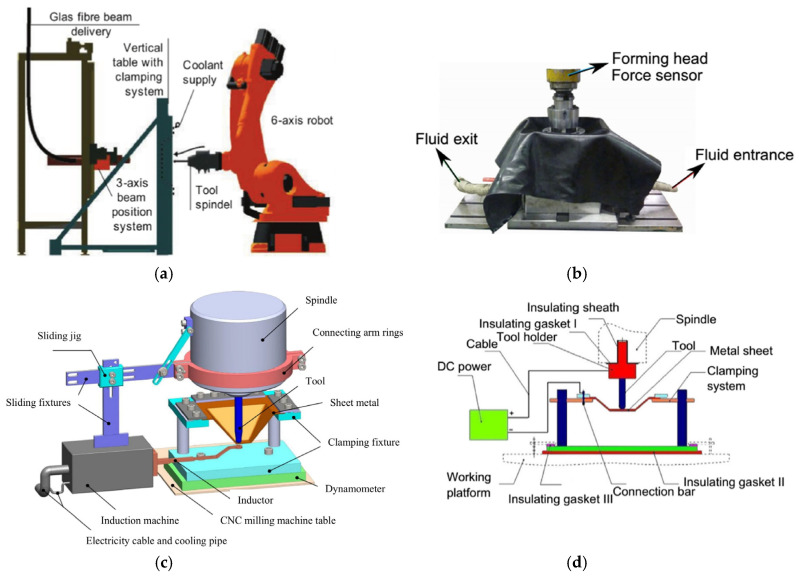
Typical hot incremental sheet forming processes: (**a**) laser incremental sheet forming [10,11], (**b**) thermal medium incremental sheet forming [12], (**c**) induction heat-assisted incremental sheet forming [13,14], and (**d**) local electric hot incremental sheet forming [15,16].

With the continuous improvement of lightweight design and green manufacturing requirements, nonferrous metals with lightweight, high corrosion resistance, and great mechanical properties, are extensively adopted in the aerospace, biomedicine, and automotive sectors, for instance, titanium, magnesium, aluminum alloys, etc. [9]. However, these materials have a common feature, namely low ductility at room temperature and fine formability at elevated temperatures. Therefore, these materials could be formed successfully using an assistant heating system. Figure 1 shows some typical hot incremental sheet forming methods, such as laser incremental sheet forming [10,11], thermal medium incremental sheet forming [12], induction heat-assisted incremental sheet forming [13,14], and electric hot incremental sheet forming [15,16]. Thermal medium incremental sheet forming can only be adopted to fabricate material (aluminum and magnesium alloys) with lower forming temperatures due to the temperature limitation of the thermal medium. Meanwhile, the three other heating methods have a wider forming range, for which Hinoa et al. [10] and Lehtinen et al. [11] proposed a regulation approach for laser irradiation to improve the formability of some materials, such as magnesium alloy, aluminum, deep-drawing steel, and copper. Meanwhile, Duflou et al. [13] proposed a local heating method executed by laser to improve the formability and accuracy of parts with 1060 aluminum. Meanwhile, Li et al. [12] adopted induction heat-assisted incremental sheet forming to fabricate Ti-6Al-4V parts, and the forming quality and the microstructure of parts were improved through process parameter optimization. However, laser-assisted heating and induction heating are not widely used in incremental forming due to the high cost of setup and maintenance. Recently, electrically assisted manufacturing (EAM), which utilizes the electroplasticity and Joule heating effect of metals to improve the formability of materials, has received great attention from researchers as a new concept. EAM is a hybrid fabricating process aiming to enhance productivity, efficiency, and quality and to decrease costs [15,16,17]. Due to low electronic energy, the thermal effect (Joule heat) is a major factor in improving the ductility of metals in EAIF. Fan et al. [18] proposed a novel local electric hot incremental forming method (Figure 1) to rapidly form parts with magnesium and titanium alloys, and then a series of studies [19,20,21,22] for this technology were carried out.

**Figure 2:**
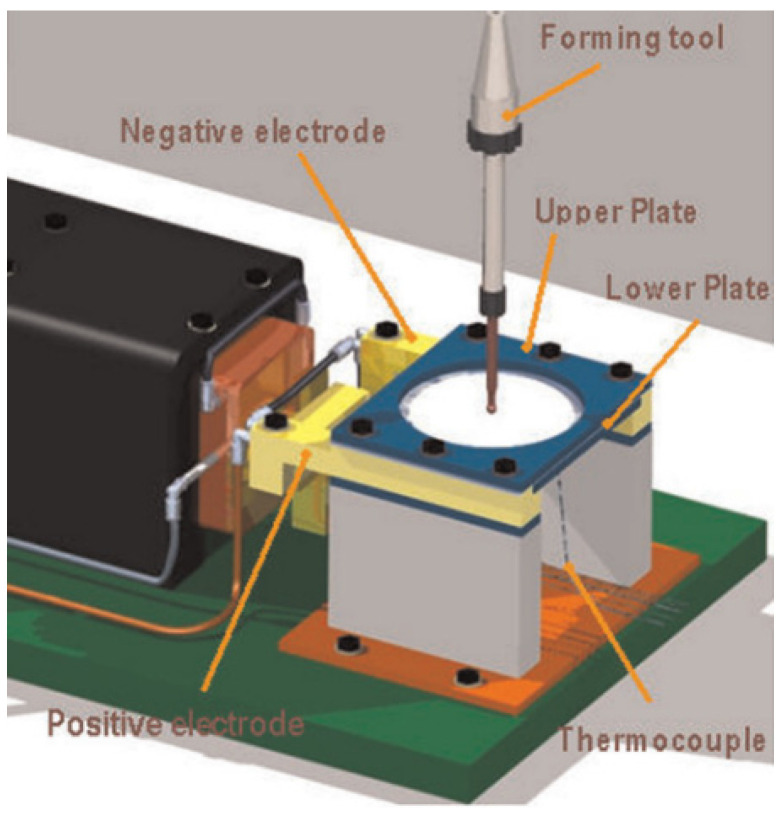
IEHIF scheme [23].

**Figure 3:**
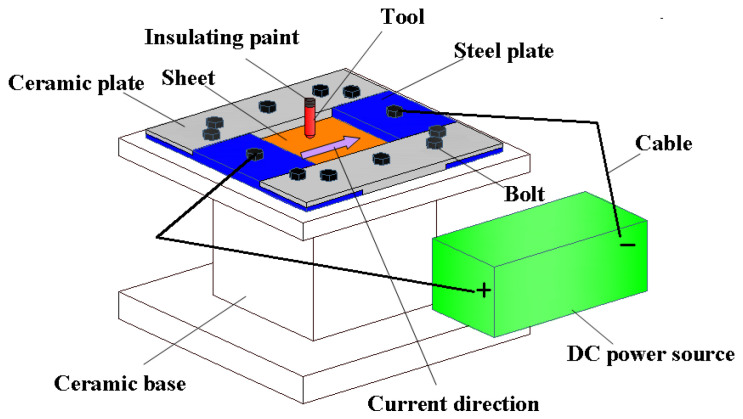
Improved scheme of IEHIF [24].

Although the local electric hot incremental forming (LEHIF) process has been adopted to fabricate small batch parts with hard-to-form materials, this process has some natural defects: inhomogeneous temperature distribution, arc burns for the sheet and the tool, unsuited for multistage forming, complex lubrication processes, etc. According to the above defects, Le Van and Nguyen [23] adopted the integral electric hot incremental forming (IEHIF) process (Figure 2), which makes current flow through the sheet metal to elevate the forming temperature of the whole plate during incremental forming to obtain a better uniform forming temperature, which is more conducive to the forming accuracy of materials. However, the clamp and the support plate of the system were made of steel without insulation. Therefore, the loss of electrical energy was fairly high due to electric shunting, and then the method could not fulfill the demand for green production in modern industry. Subsequently, Li et al. [24] further improved the design (Figure 3) of the forming device based on IEHIF to improve the utilization rate of electrical energy, and the conical box part of Ti-6Al-4V titanium alloy was also fabricated successfully.

For the moment, although the IEHIF process overcomes these natural defects obtained by LEHIF, there are still some problems that need further study, such as numerical simulation, electrode design, etc. In this work, heat flux models for electric hot tension and IEHIF of Ti-6Al-4V titanium alloy were separately established to simplify the coupled electro-thermal-mechanical analysis to the thermal–mechanical simulation based on the static Joule heat effect, which could provide accurate thermal boundary conditions for the simplified simulation. Meanwhile, the effect of the electrode structure on temperature distribution was analyzed in detail, and then a design criterion of the electrode length was obtained during IEHIF.

## 2. Materials and Methods

### 2.1. Heat Flux Models

**Figure 4:**
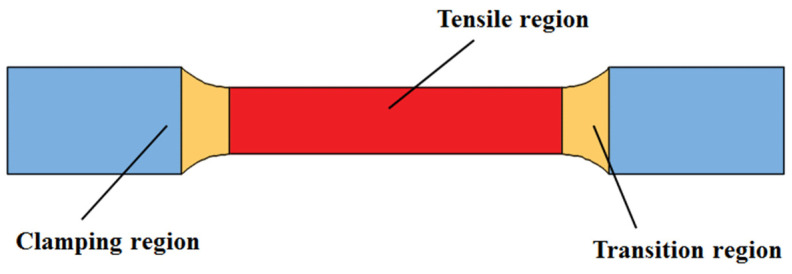
Sketch of electric hot tensile specimen.

During electric hot tension and IEHIF, the thermal source is both the Joule heat effect, and then the simplified simulation just needs to transform the coupled electro-thermal analysis into heat transfer analysis. For electric hot tensile tests, specimens are divided into three parts (Figure 4), namely a clamping region, a transition region, and a tensile region.

According to Figure 4, the clamping region contacts the electrode, and it obtains thermal energy depending on the effect of Joule heat on the contact resistance. Meanwhile, the other two regions rely on the self-resistance of each region to obtain thermal energy. Therefore, the electric power of each region is obtained:

(1) $$Q_{J}=I^{2}R_{1}={\left(J_{1}A_{1}\right)}^{2}R_{1}$$

(2) $$Q_{g}={I^{2}R}_{2}={\left(J_{1}A_{1}\right)}^{2}R_{2}$$

(3) $$Q_{C}={I^{2}R}_{C}={\left(J_{1}A_{1}\right)}^{2}R_{C}$$

where *Q_J_* is the electric power of tensile regions, *Q_g_* is the electric power of transition regions, and *Q_C_* is the electric power of clamping regions. *I* is the tensile current value, and *J*_1_ is the current density of the cross-section in the tensile regions. *R*_1_ is the resistance value of the tensile regions, *R*_2_ is the resistance value of the transition regions, and *R_C_* is the contact resistance value of the clamping regions. *A*_1_ is the sectional area of current action for tensile regions.

**Figure 5:**
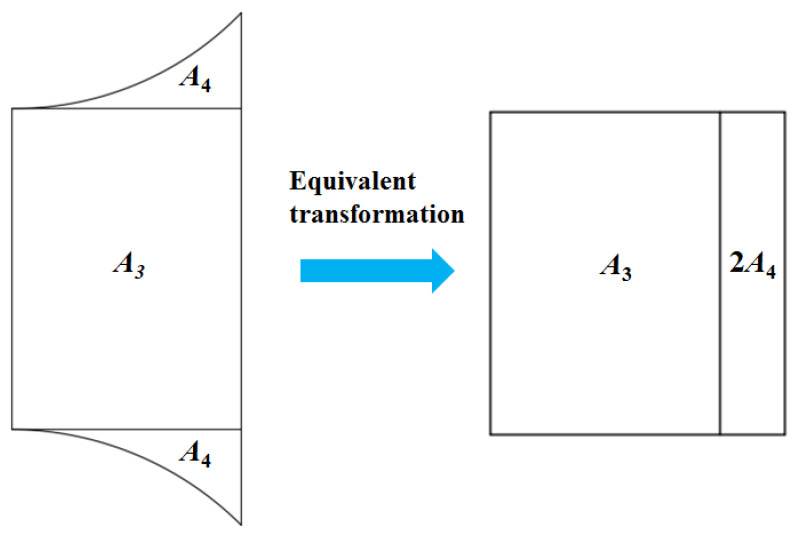
Sketch of equivalent transformation of transition region.

*R*_2_ is difficult to calculate due to the irregularity of the region, and then an equivalent transformation should be exerted to make it regular. Here, an area equivalence principle is adopted to obtain a regular shape, which is shown in Figure 5.

**Figure 6:**
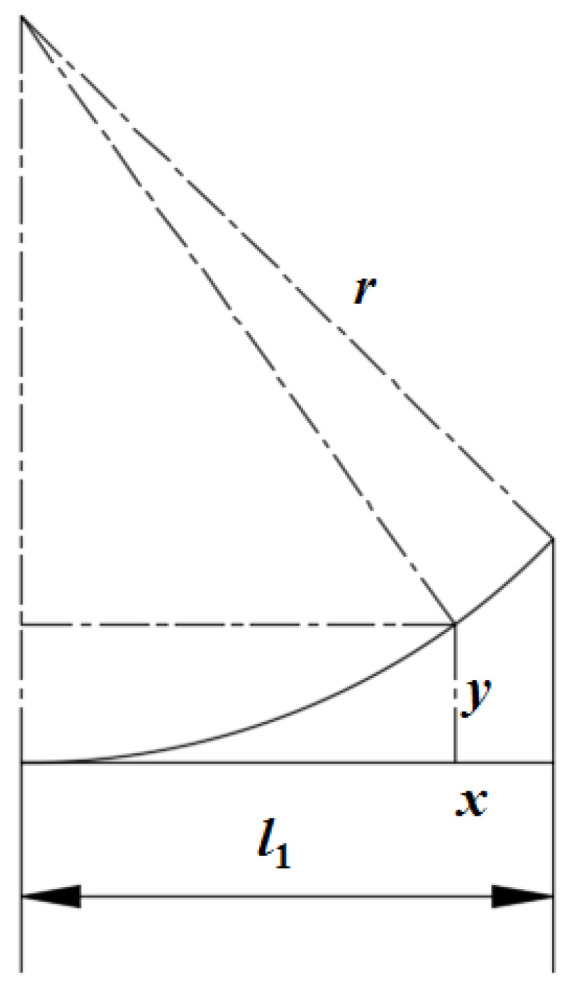
Sketch of arc region.

According to Figure 5, the essence of the equivalent exchange is that the arc region is transformed into a rectangular area. Therefore, the arc region from Figure 5 is further analyzed and it is shown in Figure 6. Based on the Pythagorean theorem and the integral rule, the area of the arc region *A*_4_ is given in Equation (4).

(4) $$A_{4}=\int_{0}^{l_{1}}r-\sqrt{r^{2}-x^{2}}dx$$

Because of a specific solution of zero, Equation (4) is further written as Equation (5) according to the integral solution law.

(5) $$A_{4}=l_{1}\left(\frac{2r-\sqrt{r^{2}-l_{1}^{2}}}{2}\right)-\frac{r^{2}}{2}\arcsin\frac{l_{1}}{r}$$

Therefore, *R*_1_ and *R*_2_ separately from Equations (1) and (2) could be obtained through the calculation rule of resistance, which is given in Equation (6):

(6) $$R_{i}=\rho (T)\frac{l}{S}=\int_{0}^{l_{j}}\frac{\rho (T)}{A_{1}}dl$$

where *i* is a subscript, and *i* = 1 or 2. *j* is a subscript, and *j* = 2 or 3. *ρ*(*T*) is the electrical resistivity related to temperature. *l*_2_ is the length of the tensile region, and *l*_3_ is the length of the transition region transformed.

The contact resistance of the clamping region is calculated according to the work of Tslaf [25] and it is obtained as follows:

(7) $$R_{C}=\rho (T)\sqrt{H(T)/H(T_{R})}$$

where *H*(*T*) is the hardness of materials at temperature *T*, and *H*(*T_R_*) is the hardness of materials at room temperature.

**Figure 7:**
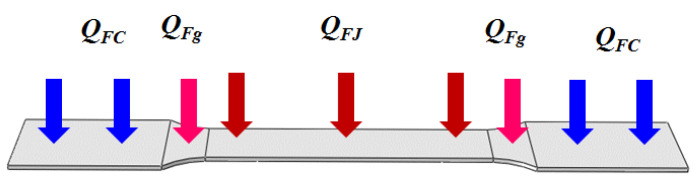
Thermal load surface of tensile specimens.

After determining the electric power of each region, the heat flux of each region is separately given in Equations (8)–(10), according to the thermal load surface of each region (Figure 7).

(8) $$Q_{FJ}=\frac{Q_{J}}{A_{J}}$$

(9) $$Q_{Fg}=\frac{Q_{g}}{A_{3}+2A_{4}}$$

(10) $$Q_{FC}=\frac{Q_{C}}{A_{C}}$$

where *Q_FJ_* is the heat flux of tensile regions, *Q_Fg_* is the heat flux of transition regions, and *Q_FC_* is the heat flux of clamping regions. *A_J_* is the area of heat flux action of the tensile regions, and *A_C_* is the area of heat flux action of the clamping regions.

**Figure 8:**
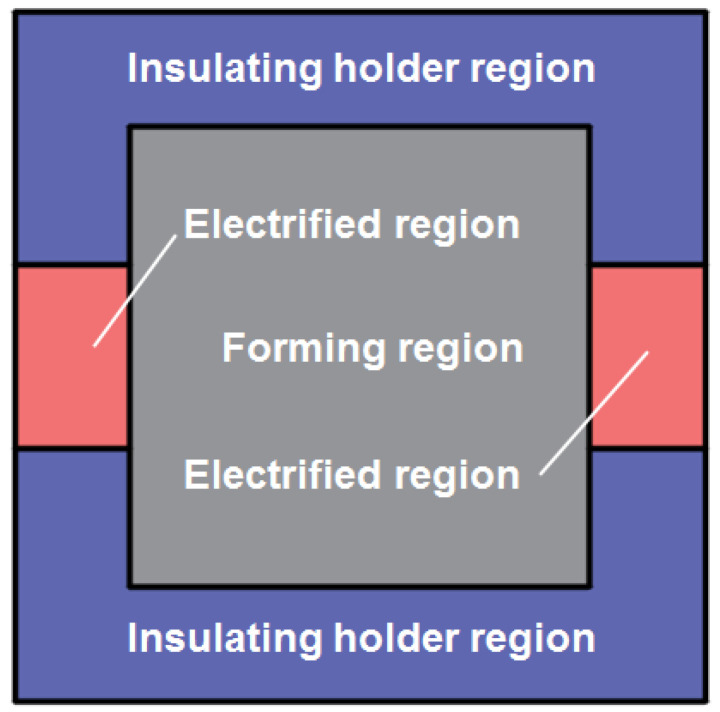
Thermal load surface during the IEHIF process.

**Figure 9:**
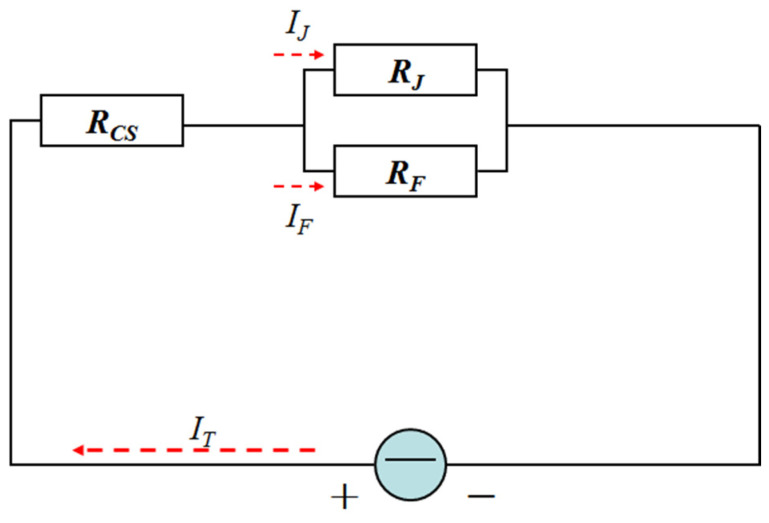
The circuit diagram of the sheet in IEHIF.

During the IEHIF process, the sheet is also divided into three fields in Figure 8, including the electrified region (ER), insulating holder region (IHR), and forming region (FR). The closed return circuit is composed of the above three regions, and then the sheet is viewed as the contact resistance of ER (RCS) being in series with the parallel resistance between the resistance of IHR (RJ) and the resistance of FR (RF), which is shown in Figure 9.

Based on the characteristics of the parallel circuit, the current of IHR (*I_J_*) and the current of FR (*I_F_*) are separately given in Equations (11) and (12):

(11) $$I_{J}=\frac{I_{T}R_{F}}{R_{J}+R_{F}}$$

(12) $$I_{F}=\frac{I_{T}R_{J}}{R_{J}+R_{F}}$$

where *I_T_* is the total current in IEHIF.

The electric power of FR (*Q*_1_), the electric power of IHR (*Q*_2_), and the electric power of ER (*Q*_3_) are, respectively, calculated referring to Equations (1)–(3). Meanwhile, the resistance value of each region is also obtained according to Equations (6) and (7), and then the heat flux of each region could be given in Equations (13)–(15), referring to heat flux models of tensile specimens:

(13) $$Q_{F1}=\frac{Q_{1}}{A_{F1}}$$

(14) $$Q_{F2}=\frac{Q_{2}}{A_{F2}}$$

(15) $$Q_{F3}=\frac{Q_{3}}{A_{F3}}$$

where *Q_F_*_1_ is the heat flux of FR, *Q_F_*_2_ is the heat flux of IHR, and *Q_F_*_3_ is the heat flux of ER. *A_F_*_1_ is the area of FR in IEHIF, *A_F_*_2_ is the area of IHR in IEHIF, and *A_F_*_3_ is the area of ER in IEHIF.

### 2.2. Design of Electrode Structure

For tensile specimens, the electrode structure mainly depends on the design of the clamping region of specimens. However, the effect of the electrode structure on the temperature distribution of FR and the resistance of IHR is significant in IEHIF, and then the electrode structure should be analyzed and designed in detail before modeling heat flux.

**Figure 10:**
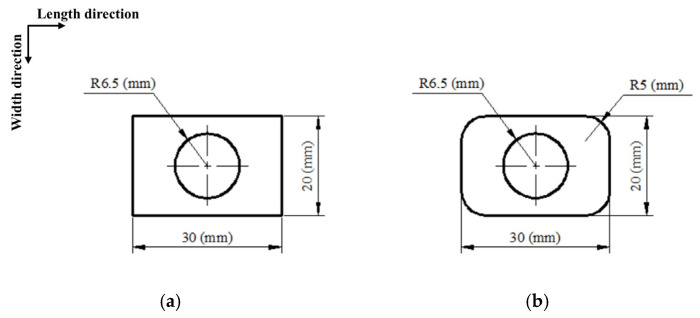

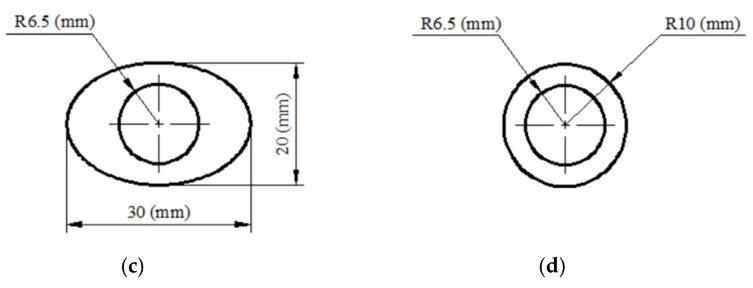
Electrode sections: (**a**) RAR, (**b**) RCR, (**c**) ellipse, and (**d**) circle.

The electrode width is constant when the blank holder area has been determined. Therefore, factors for the temperature distribution mainly include the electrode section and length. In this work, four kinds of electrode sections (Figure 10) were designed according to the study of Zhang [26] to analyze the effect of the electrode section on the temperature distribution, thereby obtaining a reasonable section. The sheet thickness was 1 (mm) with Ti-6Al-4V titanium alloy, the plane size of the sheet designed was 200 × 200 (mm), and the length of FR and the holder width were separately 160 (mm) and 20 (mm). Moreover, five length values, including 30, 60, 90, 120, and 150 (mm), were adopted based on the reasonable section, which obtained an optimal length value.

### 2.3. Finite Element Modeling

**Figure 11:**
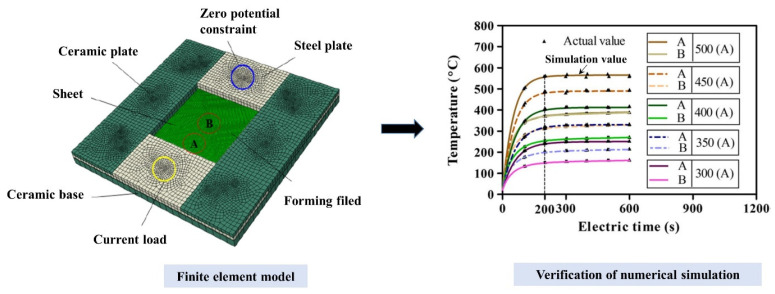
The verification for the electric–thermal simulation [24].

The two simulation methods, electric–thermal and thermal transfer simulations, were set to analyze the static effect of Joule heat and to verify the accuracy of the heat flux model. Eight-node linear coupled thermal–electrical elements were applied to the two simulations, in which the element sizes of the sheet and the electrode were 2 and 4 (mm), respectively. The accuracy of the electric–thermal simulation would be validated based on a previous study [24], which is shown in Figure 11. The materials of the sheet and the electrode were both Ti-6Al-4V titanium alloy to obtain a more uniform temperature distribution. Meanwhile, the thermal convection and radiation between the sheets and air were exerted according to Equation (16):

(16) $$Q_{R}=S_{1}(K_{1}\left(T-T_{R}\right)+\varepsilon \sigma {\left(T-T_{R}\right)}^{4})$$

where *Q_R_* is the thermal lost energy, and *S_1_* is the contact area between sheets and air. *K*_1_ is the thermal convection coefficient of air, and it was 5 × 10^−5^ (W/(mm^2^·°C)). *ε* is the radiation coefficient of Ti-6Al-4V titanium alloy, and it was 0.3. *σ* is the Boltzmann constant, and it was 5.67 × 10^−14^ (W/(mm^2^·°C^4^)).

**Figure 12:**
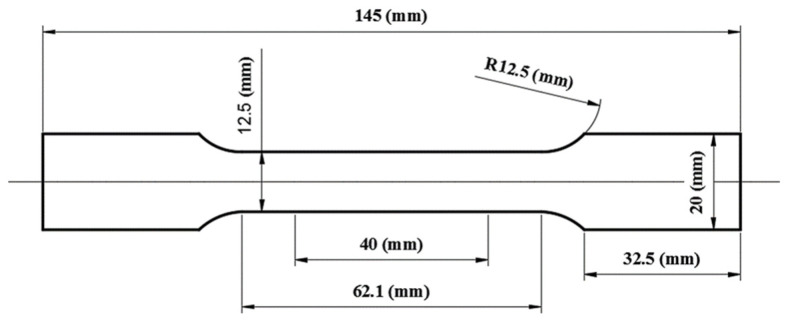
Sketch of standard tensile specimen.

The thermal transfer simulation was established based on the electric–thermal simulation, in which the electric properties of the material were ignored and the electric boundary condition was transformed into the boundary condition of surface heat flux. In the electric tensile simulation, the standard tensile specimen was designed with 1 (mm) thickness, and its dimensions are shown in Figure 12. In order to establish the relation between temperature and time, the temperature rising rate of specimens was collected every other 0.8 in the range of *J*_1_ of 0.8 to 9.6 (A/mm^2^); in addition to this, some current loads, such as 100, 200, 300, 400, 500, 600, 700, 800, 900, and 1000 (A), were separately exerted to analyze the relation between the temperature rising rates of each region of the sheet and current during the IEHIF process.

## 3. Results and Discussion

### 3.1. Analysis of Electrode Structures

**Figure 13:**
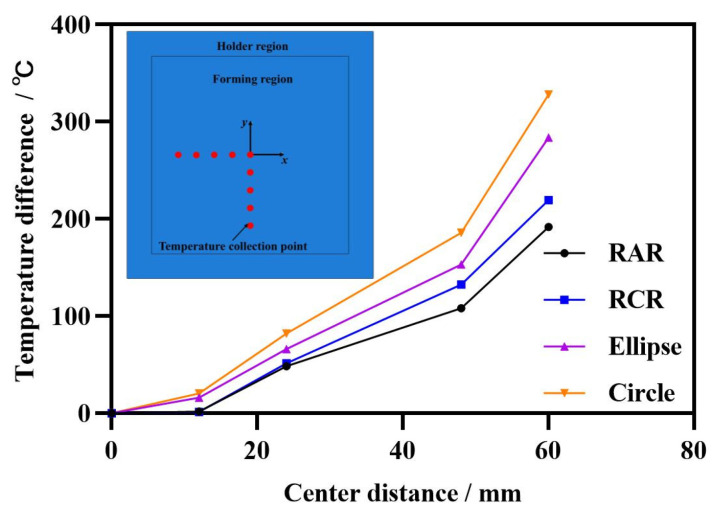
The temperature distribution of the forming region under different electrode sections.

Figure 13 shows the temperature difference between *x* and *y* directions under 500 (A) current, 300 (s) electric time, and different sections of the electrode with 20 (mm) thickness. The centers of the forming regions both obtain the temperature distribution of an hourglass, in which the temperature gradient is more significant than other conditions while adopting the circular electrode, and the temperature of each region was also relatively high, which could not contribute to enhancing the formability of the sheet metal. Therefore, the circular electrode should not be used in IEHIF. Moreover, the use of RAR could achieve better temperature uniformity compared with the use of RCR and ellipses, which would help to improve the formability of sheets. Thus, the RAR section is an optimal design and should be employed in IEHIF.

**Figure 14:**
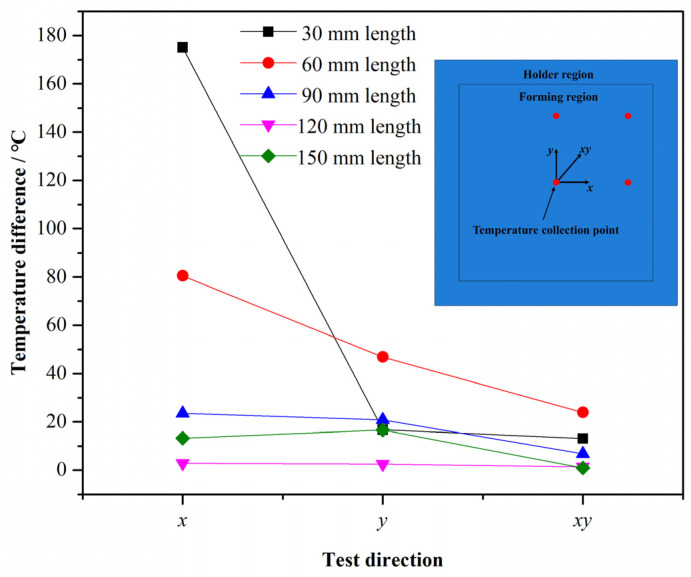
The maximum temperature difference in different directions.

**Figure 15:**
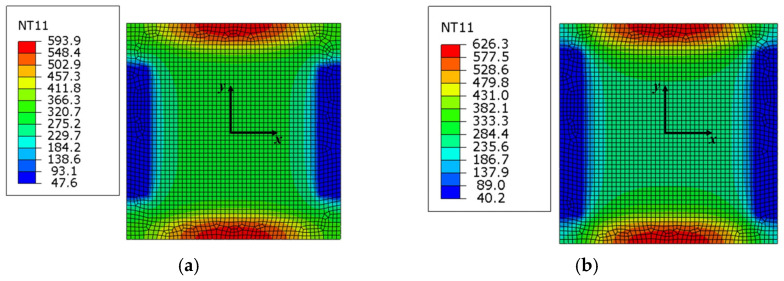
The temperature distribution of forming regions with different length values: (**a**) 120 (mm) and (**b**) 150 (mm).

For the electrode of RAR, the design scheme of the electrode length was exerted to obtain an optimal length. Figure 14 shows the maximum temperature difference in multiple directions of forming regions under different electrode lengths. In general, the temperature differences in the *x*-direction and the *y*-direction are both more than that obtained in the *xy*-direction, and the two groups, including 120 and 150 (mm), achieve smaller deviations compared with the other groups. Therefore, the two groups were further analyzed according to simulative results (Figure 15). Figure 15b shows the temperature distribution of the forming region with 150 (mm) length. Although the temperature gradient of the forming region is small, the hourglass distribution is still significant. On the contrary, the hourglass distribution of the forming region with 120 (mm) length is obviously eliminated in Figure 15a, and the temperature uniform is better than the latter one. With the above analysis, the electrode length of RAR could be calculated according to Equation (17):

(17) $$l_{e}=\frac{120}{160}l_{F}=0.75l_{F}$$

where *l_e_* is the electrode length of RAR, and *l_F_* is the length of the forming region.

### 3.2. Electrical Resistivity and Hardness

The electrical resistivity and the hardness are major factors for modeling heat flux after determining geometrical dimensions. In electric hot tension and IEHIF, the two parameters vary with temperature, and the temperature is often related to electric loads. Therefore, the temperature rising rate of different conditions should be established by the aforementioned load schemes.

**Figure 16:**
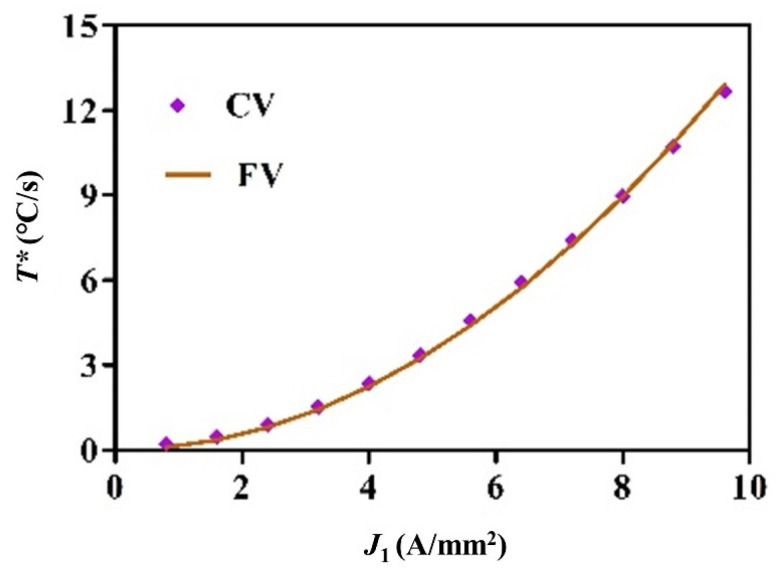
The temperature rising rate of the test area of tensile specimens.

**Figure 17:**
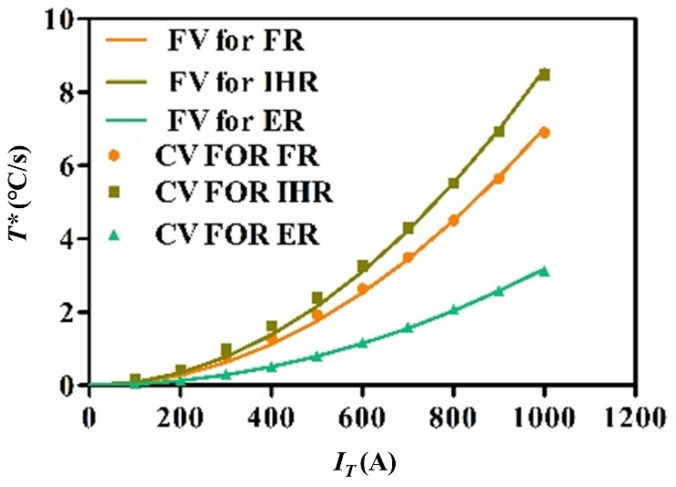
The temperature rising rate of each region of the sheet.

Figure 16 and Figure 17 separately show fitting values (FVs) and capturing values (CVs) of the temperature rising rate for electric hot tension and IEHIF, in which the collection place of tensile specimens and forming sheets is separately the tensile deformation area with 40 (mm) length and three divided regions of sheets. The temperature rising rate of each case is a parabolic distribution, and its specific solution is zero. Therefore, the temperature rising rate of each case is obtained, respectively, by Equations (18)–(21):

(18) $$T_{L}^{*}=0.14J^{2}$$

(19) $$T_{F}^{*}=7.02\times 10^{-6}{I_{T}}^{2}$$

(20) $$T_{J}^{*}=8.62\times 10^{-6}{I_{T}}^{2}$$

(21) $$T_{C}^{*}=3.18\times 10^{-6}{I_{T}}^{2}$$

where T_L_* is the temperature rising rate of tensile specimens, T_F_* is the temperature rising rate of FR, T_J_* is the temperature rising rate of IHR, and T_C_* is the temperature rising rate of ER. *J* is the current density of each region of tensile specimens.

For Ti-6Al-4V titanium alloy, the relation between electrical resistivity, hardness, and temperature was separately obtained by Fan [19,20], with a Vickers hardness of 300 at room temperature. Therefore, the electrical resistivity and the hardness, considering the temperature rising rate and the electric time, are further given in Equations (22) and (23):

(22) $$\rho (T)=-6\times 10^{-10}{\left(T^{*}t\right)}^{2}+7\times 10^{-7}T^{*}t+0.0017$$

(23) $$H(T)=-0.72\times 10^{-3}{\left(T^{*}t\right)}^{2}+0.557T^{*}t+295.6$$

where *t* is the electric time.

### 3.3. Analysis of Heat Flux Models

**Figure 18:**
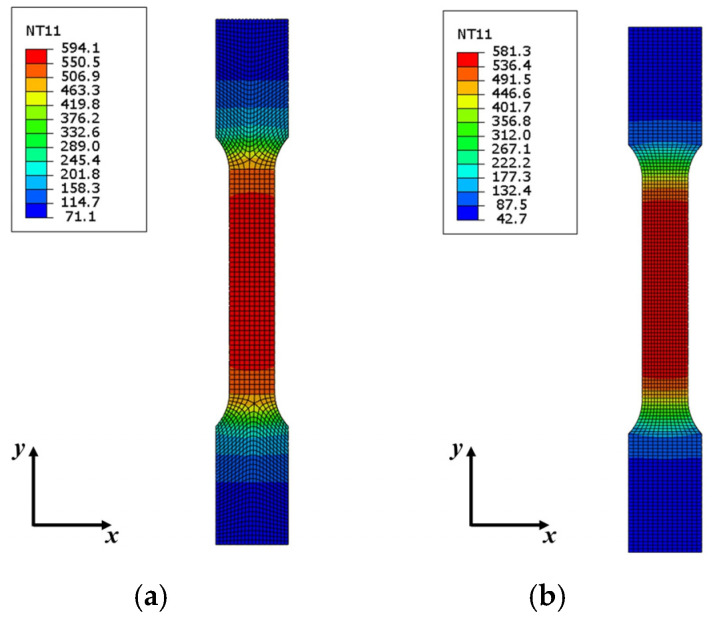
The temperature distribution of different simulations: (**a**) electro-thermal simulation and (**b**) Heat transfer simulation.

The heat flux is obtained through the above analysis when the current load and the electric time are determined. For tensile specimens, 6.4 (A/mm^2^) *J*_1_ and 120 (s) *t* were adopted to analyze the temperature distribution of the region with 12.5 × 62.1 (mm), and then the corresponding heat flux load was also gained according to the aforementioned formulae. Figure 18 shows the temperature distribution of the viewing area separately by electro-thermal and thermal transfer simulations. The center isothermal area from the electro-thermal simulation is greater than that obtained through the heat transfer simulation; on the contrary, the maximum temperature value from the heat transfer simulation is larger. Therefore, *Q_FJ_* is larger than actual electric energy, and *Q_Fg_* is small. Meanwhile, two scale factors (*p* and *q*) are introduced, where *p* is a scaling factor and *q* is a compensation factor. Thus, Equations (8) and (9) are further given in Equations (24) and (25):

(24) $$Q_{FJ}^{*}=pQ_{FJ}$$

(25) $$Q_{Fg}^{*}=qQ_{Fg}$$

Based on the energy conservation principle, *p* and *q* should conform to the following relationship:

(26) p+q=2

where *p* is assumed to be a scaling function related to the loading surface and the power loss. Therefore, considering the power loss and the ratio of current surfaces to heat flux surfaces, *p* is obtained through Equation (27):

(27) $$p=1-(\eta +\frac{A_{1}}{A_{J}+2(A_{3}+2A_{4})})$$

where *η* is the power loss factor (here, it is 0.1).

**Figure 19:**
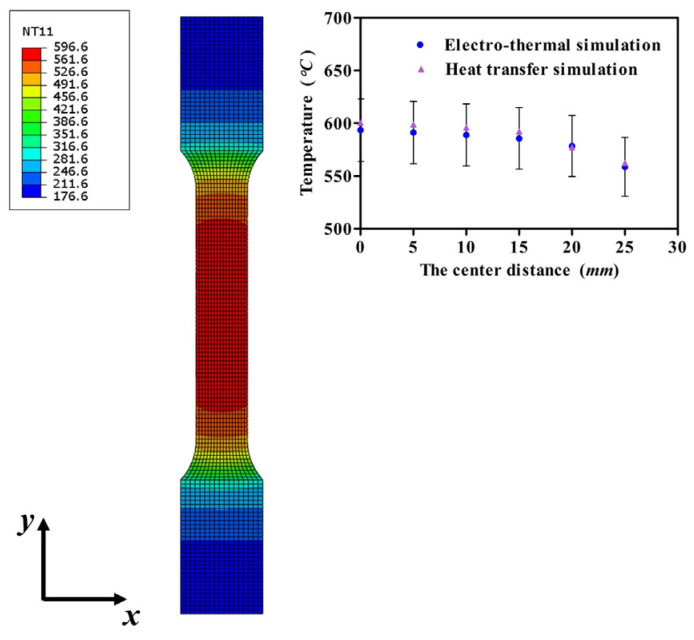
The temperature distribution of the heat transfer simulation modified and the analysis of errors between two simulations.

Through the above analysis, *p* and *q* are separately 0.888 and 1.112 according to the dimensions of tensile specimens. Figure 19 shows the modified simulation result, in which the center isothermal area from the simulation modified is similar to the result obtained by electro-thermal simulation. Meanwhile, half of the viewing area was selected to analyze the error between the two simulations, and the collection point errors are both less than 5%. Then, the assumption for *p* has been reliably verified through the previous analysis.

**Figure 20:**
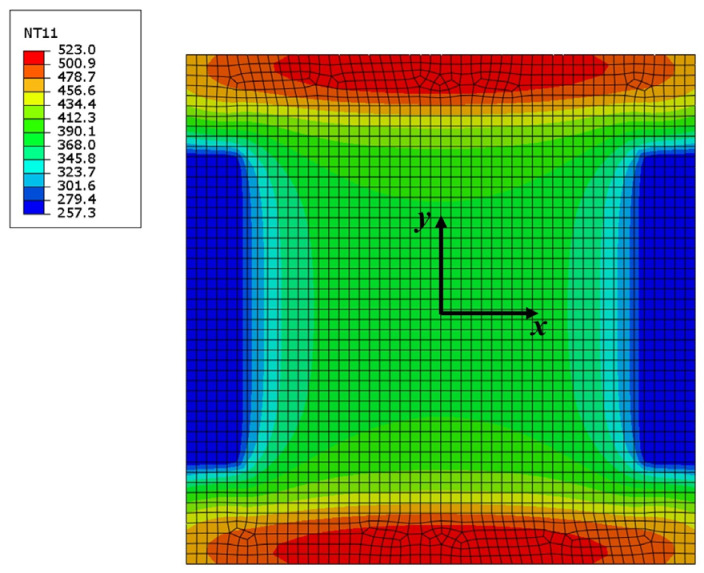
The temperature distribution of each region of the sheet with the heat transfer simulation.

In IEHIF, the heat flux model of each region is also improved by referring to the tensile condition, and the heat flux models of FR and IHR are also led into two scale factors (*p* and *q*). For the dimensions of the sheet adopted, *p* and *q* are separately 0.89 and 1.11 through Equations (26) and (27). Figure 20 shows the temperature distribution of each region of the sheet based on 500 (A) *I_T_* and 300 (s) *t*. The temperature distribution of ER and FR is similar to that obtained from Figure 15a; in addition to this, the high-temperature area of IHR is less than the result from the electro-thermal simulation. Although the difference between the temperature distributions of IHR, respectively, from electro-thermal and heat transfer simulations is significant, the region does not participate in deformation. Therefore, the temperature of the region does not produce an obvious effect on the formability of Ti-6Al-4V titanium alloy, and then the heat flux models modified are also suitable for IEHIF.

## 4. Conclusions

In general, the analysis of electrode structure offers effective guidance on the setup of IEHIF, and the heat flux model is established to simplify the coupled electro-thermal-mechanical analysis to the coupled thermal–mechanical simulation, which could successfully simulate processes of electro-thermal tension and IEHIF. The specific conclusions are as follows:

(1) The design of the electrode structure is exerted to analyze the temperature distribution of the sheet with Ti-6Al-4V titanium alloy in IEHIF, and the use of RAR could obtain a more homogeneous temperature distribution compared with the other sections.
(2) The hourglass distribution of the forming region could be eliminated when the electrode length is 0.75 times the length of the forming region. Heat flux models for electric hot tension and IEHIF are separately established based on the effect of Joule heat.
(3) Two scale factors, *p* and *q*, are proposed to correct the heat flux models of the tensile region and the transition region in electric hot tension and to improve heat flux models of FR and IHR in IEHIF, where *p* is a scaling factor and *q* is a compensation factor.
(4) The sum between *p* and *q* is 2 according to the energy conservation principle, and *p* is obtained considering the power loss and the ratio of current surfaces to heat flux surfaces.

## Data Availability

The data presented in this study are available on request from the corresponding author.
